# Anti-Inflammatory and Analgesic Effects of the Marine-Derived Compound Comaparvin Isolated from the Crinoid *Comanthus bennetti*

**DOI:** 10.3390/molecules190914667

**Published:** 2014-09-16

**Authors:** Li-Chai Chen, Yen-You Lin, Yen-Hsuan Jean, Yi Lu, Wu-Fu Chen, San-Nan Yang, Hui-Min David Wang, Ing-Yang Jang, I-Ming Chen, Jui-Hsin Su, Ping-Jyun Sung, Jyh-Horng Sheu, Zhi-Hong Wen

**Affiliations:** 1 Department of Marine Biotechnology and Resources, National Sun Yat-sen University, Kaohsiung 80424, Taiwan; E-Mails: pharmacy@mail.ngh.com.tw (L.-C.C.); chas6119@gmail.com (Y.-Y.L.); snakefoot5052@gmail.com (Y.L.); hawkiyc@gmail.com (I.-Y.J.); 99001@mail.wzu.edu.tw (I.-M.C.); sheu@mail.nsysu.edu.tw (J.-H.S.); 2 Department of Pharmacy of Zuoying Branch of Kaohsiung Armed Forces General Hospital, Kaohsiung 81342, Taiwan; 3 Section of Orthopedic Surgery, Pingtung Christian Hospital, Pingtung 90059, Taiwan; E-Mail: jean.tang@msa.hinet.net; 4 Department of Neurosurgery, Kaohsiung Chang Gung Memorial Hospital and Chang Gung University College of Medicine, Kaohsiung 83301, Taiwan; E-Mail: ma4949@cgmh.org.tw; 5 School of Medicine, College of Medicine and Department of Pediatrics, E-DA Hospital, I-Shou University, Kaohsiung 84001, Taiwan; E-Mail: y520729@gmail.com; 6 Department of Fragrance and Cosmetic Science, Center of Excellence for Environmental Medicine, Kaohsiung Medical University, Kaohsiung 80708, Taiwan; E-Mail: davidw@kmu.edu.tw; 7 General Education Center, Wenzao Ursuline University of Languages, Kaohsiung 80793, Taiwan; 8 Graduate Institute of Marine Biotechnology, National Dong Hwa University, Pingtung 94450, Taiwan; E-Mails: x2219@nmmba.gov.tw (J.-H.S.); pjsung@nmmba.gov.tw (P.-J.S.); 9 Taiwan Coral Research Center, National Museum of Marine Biology & Aquarium, Pingtung 94450, Taiwan; 10 Doctoral Degree Program in Marine Biotechnology, National Sun Yat-sen University and Academia Sinica, Kaohsiung 80424, Taiwan

**Keywords:** comaparvin, crinoids, *Comanthus bennetti*, inducible nitric oxide synthase, lipopolysaccharide, carrageenan

## Abstract

To date, no study has been conducted to explore the bioactivity of the crinoid *Comanthus bennetti*. Here we report the anti-inflammatory properties of comaparvin (5,8-dihydroxy-10-methoxy-2-propylbenzo[h]chromen-4-one) based on *in vivo* experiments. Our preliminary screening for anti-inflammatory activity revealed that the crude extract of *Comanthus bennetti* significantly inhibited the expression of pro-inflammatory proteins in lipopolysaccharide (LPS)-stimulated murine RAW 264.7 macrophage cells. Comaparvin isolated from crinoids significantly decreased the expression of inducible nitric oxide synthase (iNOS) protein and mRNA in LPS-stimulated macrophage cells. Moreover, our results showed that post-treatment with comaparvin significantly inhibited mechanical allodynia, thermal hyperalgesia and weight-bearing deficits in rats with carrageenan-induced inflammation. Comaparvin also attenuated leukocyte infiltration and iNOS protein expression in carrageenan-induced inflamed paws. These results suggest that comaparvin is a potential anti-inflammatory therapeutic agent against inflammatory pain.

## 1. Introduction

In recent years, many metabolites from marine organisms with significant potential anti-inflammatory activities have been discovered. Recently, several new medications based on substances from these marine organisms have undergone clinical and preclinical trials [1]. Natural ingredients obtained from the ocean, especially from marine invertebrates, have become important sources of new compounds [2,3,4]. Moreover, many studies have screened the anti-inflammatory properties and activities of marine compounds for the treatment of chronic illnesses [1,4,5].

In 1967, Bolker extracted and isolated a steroid compound, crinosterol, from comatulid crinoids [6]. In 1971, Smith and Sutherland isolated several quinone compounds and naphthopyrones from *Comanthus parvicirrus* [7]. The biological activities of these compounds were unclear. Research on the bioactivity of the natural products obtained from crinoids has been performed primarily *in vitro* [1,2,8,9]. Gymnochrome D and isogymnochrome D, isolated from *Gymnocrinus richeri*, have shown potential activity against dengue virus [10]. Benzochromenones obtained from *Comantheria rotula* could inhibit tumor growth through inhibition of the expression of hypoxia-inducible factor-1 (HIF-1) [11]. Tetrabromospirocyclohexadienylisoxazole compounds obtained from *Himerometra magnipinna* can inhibit *Streptomyces* [12]. The naphthopyrones isolated from *Capillaster multiradiatus* and *Comanthus parvicirrus* have been found to inhibit ABCG2 transport proteins and to prevent resistance to cancer medications [8]. The naphthopyrones comaparvin (5,8-dihydroxy-10-methoxy-2-propyl–benzo[h]chromen-4-one) and 6-methoxycomaparvin extracted from *Comanthus parvicirrus* have been shown to inhibit the signal transmission by nuclear factor-kappa B (NF-κB) [9,13], which plays an important part in the inflammatory response [14,15,16]. Numerous studies have indicated that NF-κB is a critical regulator of the expression of the pro-inflammatory protein, inducible nitric oxide synthase (iNOS) [17,18]. We found that comaparvin significantly inhibits the expression of iNOS in lipopolysaccharide (LPS)-stimulated macrophage cells. It has been demonstrated that iNOS plays a key role in the development of carrageenan-induced inflammatory responses such as paw edema and nociception [19,20]. However, studies on the *in vivo* anti-inflammatory and analgesic activity of comaparvin are few.

**Figure 1:**
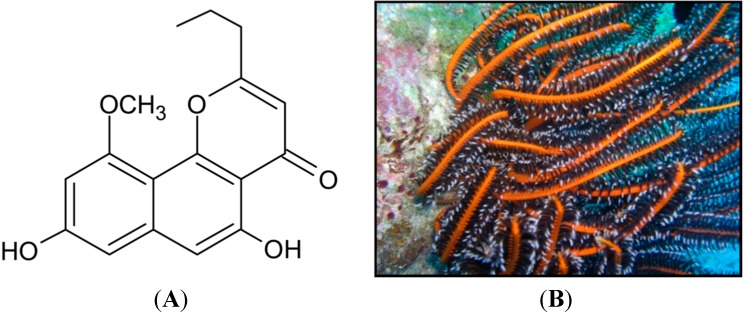
Chemical structure and source of comaparvin. (**A**) Chemical structure of comaparvin. Molecular formula, C_17_H_16_O_5_; molecular weight, 300.11 Da; (**B**) The crinoid sample, *Comanthus bennetti*, was collected from Lamay Island, Taiwan.

In the present study, we isolated comaparvin (Figure 1) from the Formosan crinoid *Comanthus bennetti*. We investigated the possible anti-inflammatory, anti-hyperalgesic, and anti-nociceptive effects of comaparvin in a carrageenan-injected rat model. Moreover, using this *in vivo* model, we also examined whether comaparvin affects the time course of the inflammatory response and the upregulation of iNOS protein expression.

## 2. Results

### 2.1. Cell Viability

**Figure 2:**
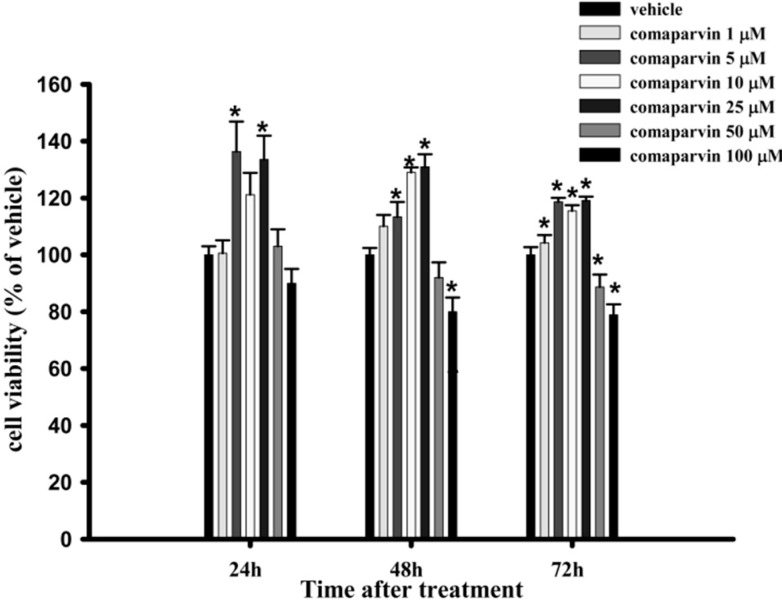
Effect of comaparvin on the viability of macrophage cells. Cells were incubated with different concentrations of comaparvin for 24, 48, and 72 h, and cell viability assessed using the alamar blue assay. Data from five independent experiments are presented as the mean ± SEM values. * *p* < 0.05 compared with vehicle groups.

To evaluate the effect of comaparvin on the viability of RAW264.7 macrophage cells, we used the alamar blue assay. The viability of macrophage cells at 24, 48, and 72 h after treatment with comaparvin (1, 5, 10, 25, 50, and 100 μM) is shown in Figure 2. Comaparvin (1 μM) did not significantly affect the viability of macrophage cells at 24, 48, and 72 h, but the viability of the cells was significantly lower when comaparvin was used at 50 μM for 72 h and at 100 μM for 48 and 72 h, as compared with the vehicle (DMSO) groups. The viability of macrophage cells treated with comaparvin at 5, 10, and 25 μM for 24, 48, and 72 h was significantly higher than that of the vehicle groups.

### 2.2. Effects of Comaparvin on LPS-Induced iNOS Protein Expression

**Figure 3:**
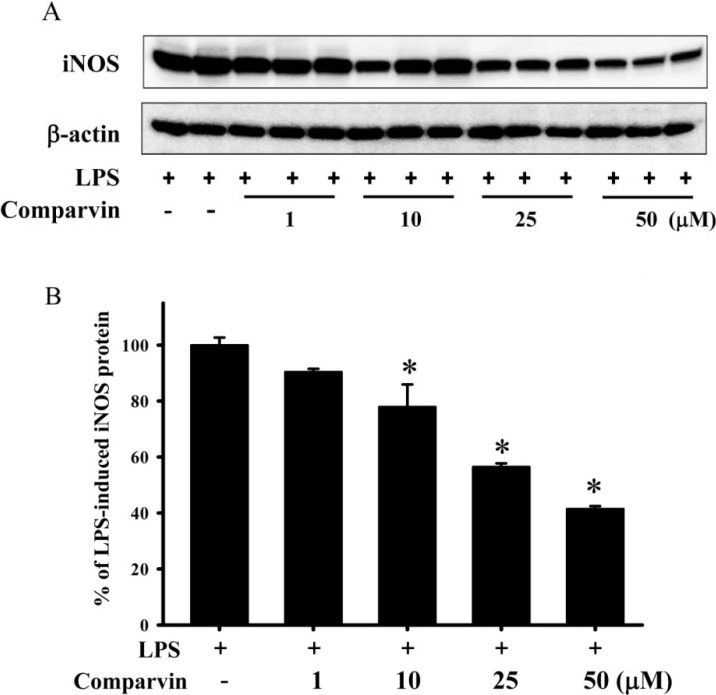
Effect of comaparvin on the expression of the pro-inflammatory protein iNOS, in LPS-stimulated macrophage cells. (**A**) Western blot bands corresponding to the effects of comaparvin on iNOS and β-actin expression in LPS-stimulated macrophage cells; (**B**) The relative intensity of expression of iNOS protein in the LPS-alone group was set to 100%, and β-actin was used to verify that equivalent amounts of protein were loaded in each lane. Comaparvin significantly inhibited iNOS protein expression in LPS-stimulated macrophage cells. Data are the mean ± SEM values of 4 independent experiments. * *p* < 0.05, significant difference compared with the LPS-alone group.

Figure 3 shows the effect of comaparvin (1, 10, 25, and 50 μM) on iNOS protein expression in LPS-stimulated macrophage cells. In the LPS-alone group, a significant increase in iNOS protein expression due to LPS challenge was noted. If LPS-induced iNOS protein expression is taken as 100%, use of comaparvin at concentrations of 1, 10, 25, and 50 μM resulted in relative iNOS protein expression of 90.42% ± 1.1%, 77.95% ± 7.99%, 56.5% ± 1.2%, and 40% ± 0.99%, respectively. Comaparvin significantly reduced LPS-induced expression of iNOS protein in macrophage cells. The β-actin protein expression was not significantly different between the different concentrations of comaparvin (1, 10, 25, and 50 μM) or from that obtained with LPS only.

### 2.3. Effects of Comaparvin on LPS-Induced iNOS mRNA Expression

**Figure 4:**
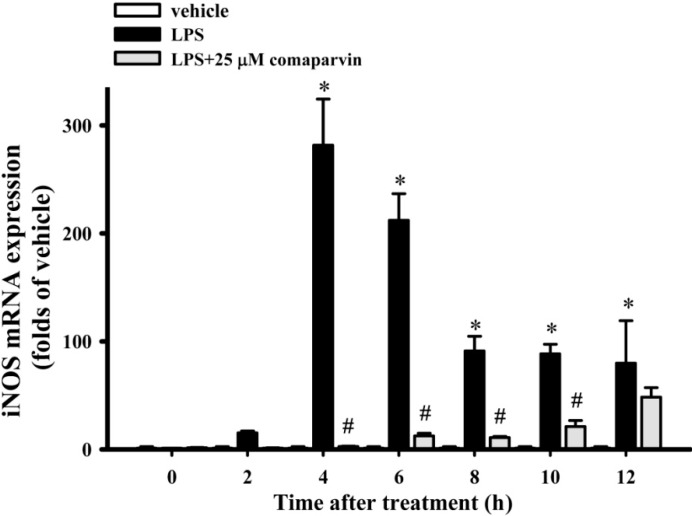
Effects of comaparvin on the expression of iNOS mRNA in LPS-stimulated macrophage cells. Cells were incubated with 25 μM comaparvin for 10 min and, then, were treated with 10 ng/mL LPS. iNOS mRNA expression was analyzed by quantitative PCR. Data are the mean ± SEM values from three independent experiments. * *p* < 0.05 compared with the vehicle groups. ^#^ *p* < 0.05 compared with the LPS-alone group.

Figure 4 shows the use of quantitative PCR to analyze the changes on iNOS mRNA expression elicited by comaparvin in LPS-induced macrophage cells. The results showed that iNOS mRNA expression at 4, 6, 8, 10, and 12 h after LPS challenge was significantly higher than that in the control group. Compared with the iNOS mRNA expression in the LPS-alone group, comaparvin at 25 μM significantly reduced iNOS mRNA expression in macrophages from 4 to 10 h. There were no significant changes in iNOS expression between time points in vehicle (no LPS challenge) group.

### 2.4. Effects of Comaparvin on Carrageenan-Induced Weight-Bearing Defects

**Figure 5:**
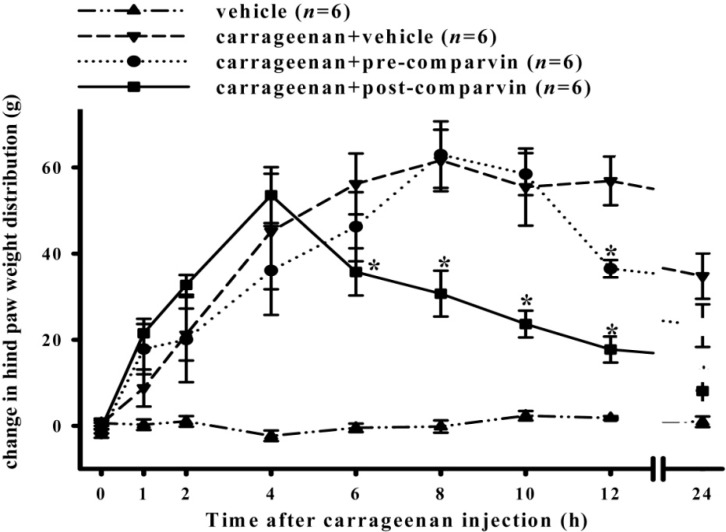
Time course of the effects of comaparvin on weight-bearing by rat hind paws after carrageenan injection. The baseline was set as the weight-bearing differential before carrageenan injection. Post-treatment of comaparvin (30 mg/kg) significantly inhibited weight-bearing defects at 6, 8, 10, 12, and 24 h after stimulation with carrageenan. Pre-comaparvin, pre-treatment of comaparvin at 1 h before carrageenan injection. Post-comaparvin, comaparvin post-treatment at 4 h after carrageenan injection. Data are the mean ± SEM values. * *p* < 0.05 compared with the carrageenan + vehicle group.

Inflammation-induced pain hypersensitivity was determined using a dual-channel weight averager (incapacitance tester) to detect the difference in the weight borne on the hind legs. Among all groups, there was no significant difference between left–right hind-paw burdens before carrageenan injection (0.45 ± 0.56 g). Figure 5 shows that, after injecting only carrageenan into the right hind paw at 4, 6, 8, 10, 12 and 24 h, the differences in left–right hind-paw weight-bearing were 45.14 ± 13.41, 56.20 ± 7.07, 61.64 ± 7.15, 55.48 ± 8.97, 56.88 ± 5.65, and 34.78 ± 5.27 g, respectively, which were significantly different from those seen in the vehicle group. In the pre-treatment group, 1 h before injecting carrageenan, a subcutaneous comaparvin injection (30 mg/kg) was administered to the rats. After injecting carrageenan, the differences in left–right hind-paw weight-bearing at 4, 6, 8, 10, 12 and 24 h were 36.08 ± 10.31, 46.28 ± 8.03, 63.00 ± 7.72, 58.48 ± 4.89, 36.50 ± 2.01, and 23.28 ± 4.95 g, respectively. In the post-treatment group, 4 h after injecting carrageenan, the rats were administered a subcutaneous comaparvin injection (30 mg/kg). The differences in left–right hind-paw weight-bearing at 4, 6, 8, 10, 12 and 24 h after injecting carrageenan were 53.58 ± 6.50, 35.76 ± 5.47, 30.70 ± 5.33, 23.64 ± 3.11, 17.72 ± 3.02, and 8.08 ± 2.95 g, respectively. For the post-treatment group, comaparvin significantly improved the defects in hind-paw weight-bearing caused by carrageenan at 6, 8, 10, 12, and 24 h. Comparison of pre-treatment and carrageenan + vehicle groups showed a significant difference only at 12 h. These results show that the post-treatment group had significant improvement of defects in hind-paw weight-bearing caused by carrageenan.

### 2.5. Effects of Comaparvin on Carrageenan-Induced Thermal Hyperalgesia

**Figure 6:**
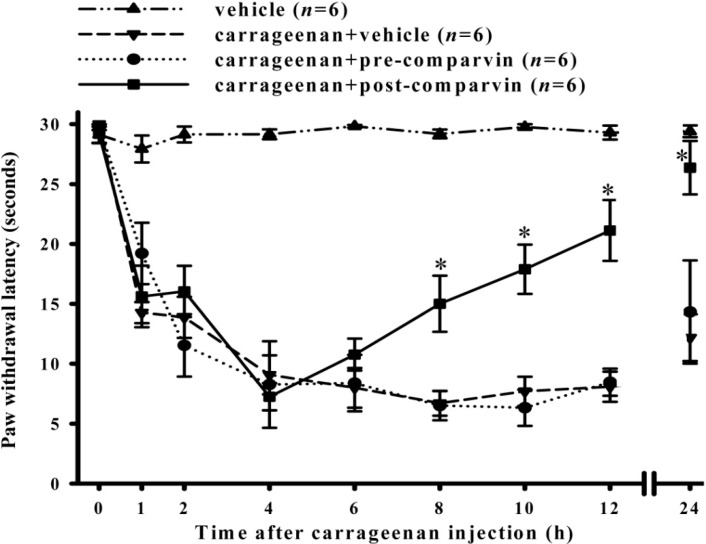
Time course showing the effects of comaparvin on carrageenan-induced thermal hyperalgesia. Comaparvin (30 mg/kg) was administered 4 h after carrageenan injection (post-comaparvin) and significantly inhibited carrageenan-induced thermal hyperalgesia at 8, 10, 12, and 24 h. Pre-comaparvin, pre-treatment of comaparvin at 1 h before carrageenan injection. Post-comaparvin, comaparvin post-treatment at 4 h after carrageenan injection. Data are the mean ± SEM values. * *p* < 0.05, significant difference compared with the carrageenan + vehicle group.

A radiant heat source was used to stimulate the central part of the hind paws to analyze the effect of comaparvin on carrageenan-induced thermal hyperalgesia. The paw withdrawal latency for the rats (before carrageenan injection) was 29.13 ± 0.67 s. For the carrageenan + vehicle group, after injecting carrageenan into the right hind paw, the paw withdrawal latencies at 1, 2, 4, 6, 8, 10, 12 and 24 h were 14.28 ± 0.9, 13.88 ± 1.7, 9.07 ± 1.63, 7.99 ± 1.66, 6.71 ± 1.04, 7.70 ± 1.22, 8.08 ± 1.26, and 12.18 ± 1.96 s, respectively, which were significantly different from those in the vehicle group (Figure 6). In the pre-treatment group, after injecting carrageenan, the paw withdrawal latencies at 1, 2, 4, 6, 8, 10, 12 and 24 h were 19.2 ± 12.6, 11.54 ± 2.6, 8.27 ± 3.61, 8.39 ± 2.35, 6.50 ± 1.22, 6.32 ± 1.51, 8.46 ± 1.14, and 14.33 ± 4.31 s, respectively. In the post-treatment group, the duration of thermal hyperalgesia at 1, 2, 4, 6, 8, 10, 12, and 24 h was 15.62 ± 2.6, 16.05 ± 2.1, 7.25 ± 1.13, 10.77 ± 1.33, 15.01 ± 2.34, 17.89 ± 2.06, 21.13 ± 2.54, and 26.37 ± 2.23 s, respectively. Paw withdrawal latency for the post-treatment group at 8, 10, and 12 h showed significant differences compared with the carrageenan + vehicle group. There were no significant differences between the pre-treatment group and carrageenan-induced group. These results show that the post-treatment group experienced improvement in carrageenan-induced thermal hyperalgesia.

### 2.6. Effects of Comaparvin on Carrageenan-Induced Mechanical Allodynia

**Figure 7:**
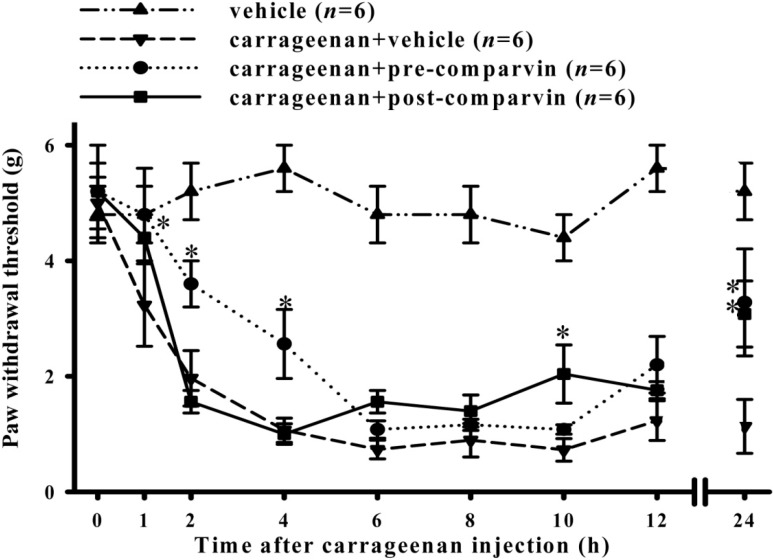
Time course showing the effects of comaparvin on carrageenan-induced mechanical allodynia. The von Frey threshold (g) decreased progressively with a maximum value at 4 to 10 h after carrageenan injection. The pre-treatment comaparvin (30 mg/kg) group showed significantly attenuated carrageenan-induced mechanical allodynia at 1, 2, 4, and 24 h, and the post-treatment group showed significantly attenuated carrageen-induced mechanical allodynia at 10 h and 24 h. Pre-comaparvin, pre-treatment of comaparvin at 1 h before carrageenan injection. Post-comaparvin, comaparvin post-treatment at 4 h after carrageenan injection. Data are the mean ± SEM values. * *p* < 0.05, significant difference compared with the carrageenan + vehicle group.

Testing using von Frey monofilaments was used to detect mechanical allodynia. The von Frey monofilament withdrawal threshold for the control group was 5.02 ± 0.17 g. In the carrageenan + vehicle group, after injecting carrageenan into the right paws, the von Frey thresholds at 1, 2, 4, 6, 8, 10, 12, and 24 h were 3.23 ± 0.7, 1.97 ± 0.5, 1.07 ± 0.21, 0.73 ± 0.16, 0.89 ± 0.29, 0.73 ± 0.19, 1.23 ± 0.34, and 1.13 ± 0.47 g, which were significantly different from those of the control group (Figure 7). For the pre-treatment group, the von Frey thresholds at 1, 2, 4, 6, 8, 10, 12 and 24 h were 4.8 ± 0.8, 3.6 ± 0.4, 2.56 ± 0.60, 1.08 ± 0.15, 1.16 ± 0.10, 1.08 ± 0.08, 2.20 ± 0.49, and 3.28 ± 0.93 g. For the post-treatment group, the von Frey thresholds at 1, 2, 4, 6, 8, 10, 12, and 24 h were 4.4 ± 0.4, 1.56 ± 0.2, 1.00 ± 0.18, 1.56 ± 0.19, 1.40 ± 0.28, 2.04 ± 0.50 g, 1.76 ± 0.15, and 3.08 ± 0.57 g. Significant improvement in the von Frey thresholds caused by carrageenan injection was seen at 1, 2, 4 and 24 h for the pre-treatment group. Significant improvement for the post-treatment group was seen only at 10 and 24 h. These results show that the pre-treatment group and post-treatment groups had improved hind-paw withdrawal thresholds due to carrageenan.

### 2.7. Effect of Comaparvin on Carrageenan-Induced Cell Infiltration in Paw Tissue

**Figure 8:**
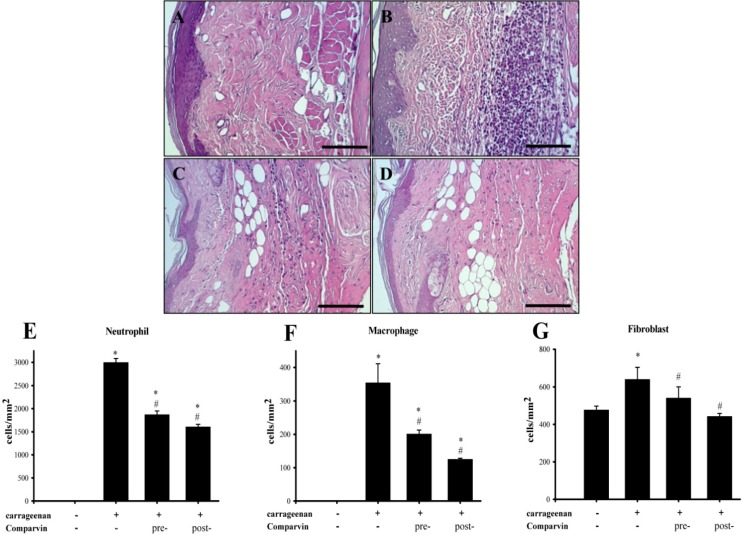
Histopathologic analyses of the effects of comaparvin on cell infiltration in carrageen-injected paws. (**A**) Vehicle group (**B**) Carrageenan-alone group (**C**) Pre-treatment group (**D**) Post-treatment group. Pre-treatment or post-treatment of comaparvin (30 mg/kg) appeared to attenuate carrageenan-induced upregulation of immune cells. Numbers of neutrophils (**E**); macrophages (**F**); and fibroblasts (**G**) were analyzed in paw tissue from each group. Pre, comaparvin pre-treatment at 1 h before carrageenan injection. Post, comaparvin post-treatment at 4 h after carrageenan injection. Scale bar = 200 μm. Data are the mean ± SEM values. * *p* < 0.05, significant difference compared with the control group. ^#^ *p* < 0.05, significant difference compared with the vehicle group.

H&E staining showed carrageenan-induced clusters of infiltrating cells in paw tissue. No neutrophils or macrophages were observed in the paw tissue of rats in the control group. The neutrophil counts in the carrageenan, pre-treatment, and post-treatment groups were 2994.5 ± 86.8, 1870 ± 89.4, and 1,606 ± 55.1 cells/mm^2^, respectively (Figure 8). The number of macrophages in the carrageenan, pre-treatment, and post-treatment groups was 354.8 ± 56.4, 201 ± 12, and 124.9 ± 3.3 cells/mm^2^, respectively. Comaparvin could effectively inhibit the expression of neutrophil and macrophage aggregates in the paw tissue. The number of fibroblasts in the control, carrageenan, pre-treatment, and post-treatment groups was 476.9 ± 20.5, 640.37 ± 63, 540.7 ± 59.7, and 442.8 ± 15 cells/mm^2^, respectively. Comaparvin could effectively inhibit fibroblast expression in paw tissue.

### 2.8. Effects of Comaparvin on Carrageenan-Induced iNOS Expression in Inflamed Paw Tissue

**Figure 9:**
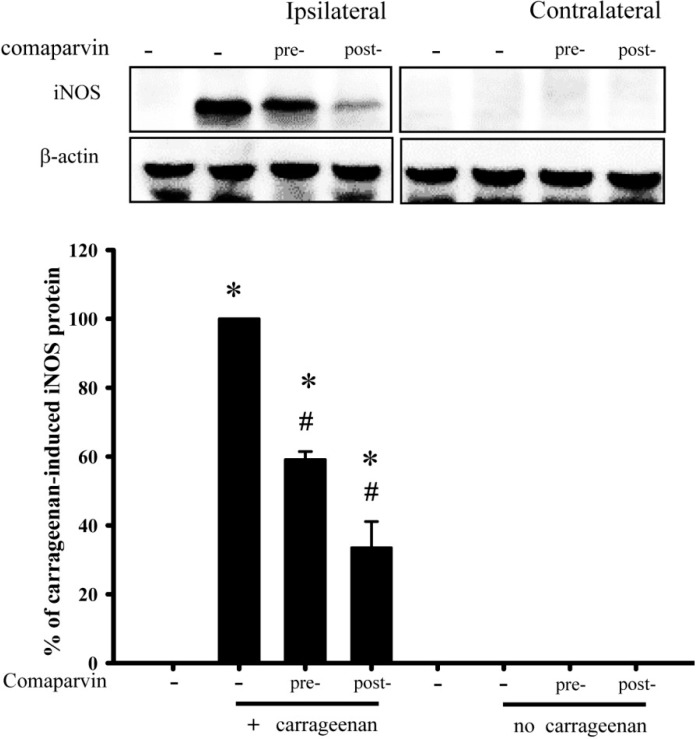
Inhibitory effect of comaparvin on the expression of carrageenan-induced iNOS protein expression in rat paw tissue. Western blot analyses of expression of iNOS and β-actin protein in paw tissues after carrageenan injection. Intraplantar carrageenan injection induced significant iNOS protein expression in ipsilateral (carrageenan injection site) but not contralateral paw tissues at 24 h. Administration of a subcutaneous injection of comaparvin (30 mg/kg) significantly inhibited carrageenan-induced upregulation of iNOS protein expression. β-actin protein expression remained unchanged in all groups. Quantification data show the mean ± SEM values for four different experiments. Pre, comaparvin pre-treatment at 1 h before carrageenan injection. Post, comaparvin post-treatment at 4 h after carrageenan injection. Data are the mean ± SEM values. * *p* < 0.05 compared with the vehicle group. ^#^ *p* < 0.05 compared with the carrageenan group.

Western blot analyses were used to detect iNOS expression in hind paw tissue. Rats were sacrificed 24 h after carrageenan injection and tissue was collected from the ipsilateral paw (right paw) and contralateral paw (left paw) (Figure 9). There was no significant iNOS protein expression in the ipsilateral paw tissue of the control group. After carrageenan injection, iNOS protein expression was considered 100% in the carrageenan group, and the relative iNOS protein expression in the pre-treatment and post-treatment groups was 59.1% ± 2.3% and 33.52% ± 7.6%, respectively. There was no significant iNOS protein expression in tissue in the contralateral paw. Pre-treatment and post-treatment with comaparvin both inhibited carrageenan-induced iNOS protein expression in paws.

## 3. Discussion

Folmer *et al.* [13] isolated naphthopyrone compounds from *Comanthus parvicirrus* and used these in *in vitro* experiments to show that these compounds can inhibit tumor necrosis factor (TNF)-α-induced NF-κB activation and the inflammatory response by I kappa B kinase (IKK) inhibition. Chovolou *et al.* found that comaparvin could inhibit TNF-α induced NF-κB activation in cancer cells [9]. Several studies have shown that upregulation of NF-κB expression plays an important role in inflammatory responses by regulation of iNOS expression [14,21]. iNOS produces large amounts of NO that can activate immune cells in inflamed tissue, thereby accelerating pathological changes [22,23]. Moreover, the increased production of NO via iNOS is associated with many disorders including cancer [21,24]. However, no study has directly examined the anti-inflammatory activity of crinoid-derived compounds *in vivo* and *in vitro*. We extracted comaparvin from *Comanthus bennetti* from the coastal region of Lamay Island, Taiwan. We used lipopolysaccharide (LPS) to stimulate macrophages as an *in vitro* model of inflammation as well as a carrageenan-induced paw inflammation model *in vivo* to evaluate the anti-inflammatory activity of comaparvin. In our present study, comaparvin significantly inhibited iNOS mRNA and protein expression with a dose-dependent effect in LPS-stimulated macrophages. iNOS has also been reported to be involved in COX-2 regulation and plays an important role in the expression of inflammatory factors [25,26]. Comaparvin did not inhibit cyclooxygenase-2 (COX-2) expression in the present study (data not shown). Hence, we suggest that the comaparvin-induced anti-inflammatory effects observed occur via iNOS expression, but not via COX-2 expression.

The carrageenan-induced paw edema model in rats is a well-established model for screening anti-inflammatory and analgesic compounds [27,28]. iNOS plays an important pathophysiological role in carrageenan-induced inflammatory responses [25,29]. Our previous study [29] used the rat model of carrageenan-induced paw edema to show that the anti-LPS-induced iNOS properties of lemnalol, extracted from the soft coral *Lemnalia cervicorni*, can also reduce carrageenan-induced expression of pro-inflammatory iNOS protein, leukocyte infiltration in inflamed tissue, and thermal hyperalgesia, thereby inhibiting paw edema. Moreover, Huang *et al.* [25] found that sinularin obtained from the soft coral *Sinularia querciformis* inhibits LPS-induced upregulation of iNOS expression in macrophages and attenuates carrageenan-induced inflammatory responses in rats. Radhika *et al.* [30] analyzed carrageenan-induced edema in rat paws to show that purified marine compounds from soft corals (*Sinularia crassa* and *Lobophytum* sp.) can reduce inflammation and inhibit paw edema. In this study, we utilized pre-treatment and post-treatment subcutaneous injections of 30 mg/kg comaparvin to analyze the *in vivo* anti-inflammatory effects. The carrageenan control group confirmed pain induction after carrageenan injection that peaked at 4 h and persisted for 24 h. We found that the post-treatment group could reduce weight-bearing deficits and thermal hyperalgesia more than the pre-treatment group. However, the pre-treatment group could effectively inhibit mechanical allodynia more than the post-treatment group before the peak of inflammation. Our results showed that comaparvin could effectively reduce inflammation and pain in the carrageenan-induced rat model.

Studies have shown that after injecting carrageenan into rat paws to induce tissue inflammation, neutrophils and macrophages gather at the inflammation site and induce cellular infiltration [25,31,32]. Neutrophils and macrophages mediate inflammatory responses in many diseases by expressing pro-inflammatory proteins that promote tissue inflammation [33]. Ribeiro *et al.* [34] showed that administration of xylopodium extracted from *Macrosiphonia velame* could reduce leucocyte infiltration in carrageenan-induced pleuritis in rats. In a previous study, we demonstrated a large degree of leucocyte infiltration in carrageenan-inflamed rat paw tissue [25]. In the present study, we used H&E staining to analyze sections of inflamed paw tissue and conducted quantitative analyses on neutrophil, macrophage, and fibroblast infiltration. In the carrageenan control group, we observed neutrophil, macrophage, and fibroblast infiltration in the paw tissue. Results showed that pre-treatment and post-treatment with comaparvin reduced the number of neutrophils, macrophages, and fibroblasts in the carrageenan-inflamed rat paw tissues. Hence, we proposed that comaparvin improves peripheral inflammatory responses by inhibition of inflammatory cell infiltration.

Marine resources have become an emerging source of natural products [3]. Several new medications based upon marine compounds are undergoing clinical trials [1]. However, the biological activity of many marine compounds is still unclear [35]. We extracted, purified, and separated the natural product comaparvin from *Comanthus bennetti*. LPS was then used to stimulate macrophages in an *in vitro* model of inflammation to evaluate the anti-inflammatory properties of comaparvin. Analyses of a carrageenan-injected rat model with respect to nociceptive behaviors, histological analysis, and western blotting showed that comaparvin, which possesses anti-inflammatory and analgesic activity, demonstrated efficacy when screened in this model. We also showed that these experimental models could be used to screen other potential anti-inflammatory and analgesic secondary metabolites from marine organisms. This is of great benefit for the screening of anti-inflammatory and analgesic compounds from marine organisms.

## 4. Experimental Section

### 4.1. Extraction of Comaparvin

In the present study, comaparvin (Figure 1A) was isolated from the formosan crinoid *Comanthus bennetti*, which was collected from Lamay Island, Taiwan (Figure 1B). Comaparvin was extracted using a standard procedure. Briefly, crinoids were minced and were extracted with 8 L of methanol–dichloromethane (1:1). The organic extract was partitioned to an aqueous suspension and was partitioned further between *n*-hexane, dichloromethane, *n*-butanol, and H_2_O. The *n*-hexane layer was separated over a normal-phase silica gel by column chromatography and was eluted with *n*-hexane, ethyl acetate, and methanol to yield 32 fractions. Fraction 24 was purified further by Sephadex LH-20 gel eluted with *n*-hexane–dichloromethane (1:4) to acquire the pure compound, comaparvin. The structure of comaparvin and its purity (>98%) were confirmed by ^1^H-NMR (400 MHz) and ^13^C-NMR (100 MHz) using a Mercury Plus 400 FT-NMR instrument (Varian Medical Systems, Palo Alto, CA, USA). Furthermore, the structure was compared with that reported in studies on natural compounds derived from crinoids [7,13].

### 4.2. Anti-Inflammatory Assay in Vitro

#### 4.2.1. Cell Culture

The method used to assess anti-inflammatory activity was modified from that of Ho *et al.* [36], Park *et al.* [37] and our previous studies [25,38]. Murine RAW 264.7 macrophages cells were obtained from the American Type Culture Collection (TIB-71; Manassas, VA, USA) and were cultured in Dulbecco’s modified Eagle’s medium (DMEM) supplemented with 10% heat-inactivated fetal bovine serum (FBS), 2 mM glutamine, 1 mM pyruvate, 4.5 g/L glucose, 50 U/mL penicillin, and 50 μg/mL streptomycin at 37 °C in a humidified incubator with 5% CO_2_:95% air under standard conditions. Inflammation in macrophages was induced by incubating them for 16 h in a medium containing 0.01 μg/mL LPS (0.01 μg/mL; Sigma-Aldrich, St. Louis, MO, USA) alone. We examined the effects of comaparvin on pro-inflammatory iNOS protein and gene expression in LPS-stimulated macrophage cells. The comaparvin (1, 10, 25, or 50 μM) was added to cells 10 min before LPS challenge. Cells then were then washed with ice-cold phosphate-buffered saline (PBS), lysed in ice-cold lysis buffer (50 mM Tris, pH 7.5, 150 mM NaCl, 1% Triton X-100, 100 μg/mL phenylmethylsulfonyl fluoride (PMSF), 1 μg/mL aprotinin), and centrifuged at 2000 *g* for 10 min at 4 °C for the anti-inflammatory assay *in vitro*. Cell viability was determined after treatment with alamar blue (Invitrogen, Carlsbad, CA, USA), a tetrazolium dye that is reduced by living cells to a fluorescent product. This assay is similar in principle to the cell viability assay using 3-(4,5-dimethyldiazol-2-yl)-2,5-diphenyltetrazolium bromide and has been validated as an accurate measure of the survival of RAW264.7 macrophage cells [39]. In cell culture experiments, comaparvin was dissolved in 100% dimethyl sulfoxide (DMSO) (clear). The final concentration of DMSO in the culture medium was 0.1%. The vehicle in the final culture medium was 0.1% DMSO.

#### 4.2.2. Western Blotting for iNOS

Western blotting was carried out as described in our previous studies [25,29]. Cell pellets were collected by washing with ice-cold PBS and were lysed in 4 °C lysis buffer (50 mM Tris, pH 7.5, 150 mM NaCl, 1% Triton X-100, 100 μg/mL PMSF, 1 μg/mL aprotinin). After centrifugation at 20,000× *g* for 30 min at 4 °C, the supernatant was obtained from the pellet. Protein concentrations were determined using a DC Protein Assay kit (Bio-Rad, Hercules, CA, USA) modified from the method of Lowry *et al.* [40]. An equal volume of sample buffer (2% sodium dodecyl sulfate (SDS), 10% glycerol, 0.1% bromophenol blue, 2% 2-mercaptoethanol, and 50 mM Tris–HCl, pH 7.2) was added to the sample, which then was loaded onto a tricine SDS-polyacrylamide (7% or 10%) gel and was electrophoresed at 80 V for 150 min. Proteins were transferred to polyvinylidene difluoride (PVDF) membranes (Immobilon-P; pore size, 0.45 μM; Millipore, Bedford, MA, USA) at 135 mA overnight at 4 °C in transfer buffer (50 mM Tris–HCl, 380 mM glycine, 1% SDS, 20% methanol). Membranes were blocked for 45 min at room temperature with 5% non-fat dry milk in Tris-buffered saline with Tween 20 (TTBS; 0.1% Tween 20, 20 mM Tris–HCl, pH 7.4, 137 mM NaCl) and then were incubated for 2 h at 37 °C with antibodies against iNOS (polyclonal antibody; 1:1500 dilution; BD Pharmingen, San Diego, CA, USA) and β-actin (monoclonal antibody; 1:1500 dilution; Sigma-Aldrich, St. Louis, MO, USA) proteins. Bands for iNOS and β-actin antibodies were recognized at ~135, ~72, and ~45 kDa, respectively. Immunoreactive bands were visualized by enhanced chemiluminescence (ECL kit; Millipore) and the BioChemi Imaging System and relative densitometric quantification was performed using LabWorks v6.2 (UVP, Upland, CA, USA). The analysis of iNOS protein expression in the *in vitro* and *in vivo* studies was carried out with 90 and 180 μg of protein per lane, respectively. The relative intensity of expression of iNOS protein in the LPS-alone group was set to 100%, and β-actin was used to verify that equivalent amounts of protein were loaded in each lane. The experiment was repeated 4 times.

#### 4.2.3. Quantitative Polymerase Chain Reaction (qPCR) Analyses for iNOS mRNA in LPS-Stimulated RAW Cells

The method used for qPCR was modified from that of Livak and Schmittgen [41] and De Gois *et al.* [42]. Cell pellets were collected into centrifuge tubes and were centrifuged at 3000× *g* for 8 min at 4 °C. Total RNA was isolated using ZR RNA MiniPrep (Zymo Research, Orange, CA, USA) according to manufacturer instructions. RNA (1 g) was reverse-transcribed using an iScript cDNA Synthesis kit (Bio-Rad, Hercules, CA, USA). Reactions were set up in duplicates in a total volume of 50 μL with 0.5 μL of each primer (final concentration, 0.2 μM), 25 μL of iQ SYBR Green Supermix (100 mM KCl, 40 mM Tris-HCl, pH 8.4; 0.4 mM of each dNTP, iTaq DNA polymerase, 50 units/mL; 6 mM MgCl_2_; SYBR Green I, 20 nM fluorescein, stabilizer; Bio-Rad) and 2.5 μL of template. The PCR program was as follows: 95 °C for 10 min and 40 cycles each of 95 °C for 15 s and 60 °C for 1 min. Melt curve analyses were carried out at the end of each experiment to verify that a single product was amplified per primer pair. Amplification and analyses were carried out using the CFX96 Touch^TM^ Real-time PCR detection system. Samples were compared using the relative CT method. The fold increase or decrease was determined relative to a blank control after normalizing to a housekeeping gene using 2^−ΔΔCT^ [41,42]. The real-time PCR oligonucleotide primers used for genotyping (forward and reverse, respectively) were as follows: iNOS, 5'-GCTGTTAGAGACACTTCTGAG-3' and 5'-CACTTTGGTAGGATTTGACTTTG-3'; β-actin, 5'-GCTTCTTTGCAGCTCCTTC-3' and 5'-GACCAGCGCAGCGA TATC-3'.

### 4.3. Carrageenan-Induced Inflammation and Pain

#### 4.3.1. Preparation of Animals

Wistar rats (weight: 250–285 g) were obtained from LASCO (Taipei, Taiwan) and each animal was used only once. Rats were maintained in Plexiglas cages within a temperature-controlled (22 °C ± 1 °C) room with a 12-h light–dark cycle and were given free access to food and water. All comaparvin and carrageenan injections were administered under 2% isoflurane anesthesia. The animal experiments were performed as per the *Guiding Principles in the Care and Use of Animals* of the American Physiology Society and were approved by Animal Care and Use Committee of National Sun Yat-sen University (Kaohsiung, Taiwan). Every effort was made to minimize the number of animals used and their suffering. 

#### 4.3.2. Design of Animal Experiments

Male Wistar rats (*n* = 24) were divided randomly equally into four groups: vehicle; 1.5% carrageenan + vehicle; 1.5% carrageenan + comaparvin (30 mg/kg) pre-treatment (pre-treatment with comaparvin 1 h before injection of carrageenan); 1.5% carrageenan + comaparvin (30 mg/kg) post-treatment (comaparvin was administered 4 h after carrageenan injection). Comaparvin was dissolved in 20% DMSO (suspension) and was delivered in a volume of 0.5 mL for subcutaneous injection of rats. The vehicle was 0.5 mL of 20% DMSO. Inflammatory responses were induced by intraplantar injection of 1.5% carrageenan lambda (Sigma-Aldrich, St. Louis, MO, USA) in 100 μL of saline into the right hind paw [25]. Pain behaviors were measured by Y.Y. Lin and L.C. Chen, who were blinded to the treatment groups.

#### 4.3.3. Analyses of the Pain Behavior of Rats

##### Weight-Bearing 

Rats were placed on an a Dual-channel Weight Averager (Singa Technology, Taoyuan, Taiwan) with their hind paws centered on two force transducers to measure the weight distribution between the hind paw, as described in our previous study [25]. Under normal conditions, naïve rats distribute their weight equally between both hind paws. However, following induction of limb inflammation, rats redistribute their weight to reduce the weight applied to the affected limb [25]. Changes in the weight distribution of the hind paws (in g) have been expressed as the difference between the affected paw and the normal paw, measured at an identical time point. Two or three recordings were taken for each rat to determine the average measurement at each time point.

##### Plantar Thermal Hyperalgesia

Thermal hyperalgesia was evaluated by placing the hind paw on a radiant heat source and measuring paw withdrawal latency at low-intensity heat with a cutoff time of 30 s (active intensity = 25) using an analgesiometer (IITC, Woodland Hills, CA, USA). Paw withdrawal latency was assessed as described previously by Hargreaves *et al.* [43] and in our previous study [25] as the average of three measurements per paw. Plantar test values of each hind paw were measured before and after injection of carrageenan at 2 h intervals from 0 h to 12 h, and at 24 h.

##### Mechanical Allodynia

To assess mechanical allodynia, we measured hind paw withdrawal thresholds using calibrated von Frey filaments (Stoelting, Wood Dale, IL, USA). Rats were placed in compartments of clear plastic cages on top of an elevated metal mesh floor, permitting easy access to paws. A series of von Frey filaments of logarithmically incremental stiffness was applied to the mid-plantar region of the hind paw from below the mesh floor using Chaplan’s “up–down’’ method involving alternate larger and smaller fibers to determine the closest filament to the threshold of pain response (licking or withdrawal), as described previously by Chaplan *et al.* [44] and in our previous study [25].

### 4.4. Preparation of Paw Tissues for Western Blot Analysis

After the final determination of paw edema (24 h after carrageenan injection), rats were sacrificed and paw samples were collected for western blotting. Tissues from the paws of the naïve, carrageenan, carrageman + comaparvin pre-treatment, and carrageman + comaparvin post-treatment groups were washed with ice-cold PBS and werehomogenized in ice-cold lysis buffer (50 mM Tris, pH 7.5, 150 mM NaCl, 1% Triton X-100, 100 μg/mL phenylmethylsulfonyl fluoride, 1 μg/mL aprotinin) using a Polytron homogenizer (5 cycles of 10 s at 3000 rpm) (Precellys^®^24, tissue homogenizer; Bertin Technologies, Aix En Provence, France). After centrifugation at 65,000× *g* for 60 min at 4 °C, the protein content of the supernatants was measured using the DC Protein Assay kit (Bio-Rad). Protein samples were ready for western blot analysis of iNOS and β-actin in paw tissue.

### 4.5. Histopathologic Analyses

For histopathologic examination, we modified the method from our previous studies [25,29]. Rats were sacrificed with PBS and 4% paraformaldehyde 24 h after carrageenan injection. Paws were resected *en bloc* and were fixed in 10% neutral formalin for 4 d. Paws were decalcified with 12.5% ethylenediaminetetraacetic acid (EDTA) and 10% neutral formalin for 3 weeks and then were sectioned on the sagittal plane through the center of samples. Specimens were dehydrated in a graded series of alcohol and were embedded in paraffin. Sections (thickness, 2 μm) were prepared for hematoxylin and eosin (H&E) staining for assessment of the general and pathologic changes in morphology under light microscopy (DM 6000B; Leica, Wetzlar, Germany) using a microscope digital image output system (SPOT Idea; Diagnostic Instruments, Sterling Heights, MI, USA).

### 4.6. Statistical Analyses

Data have been provided as mean ± SEM values. For immunoreactivity data, the intensity of each test band was expressed as the integrated optical density (IOD) calculated with respect to the average optical density of the corresponding control (LPS alone) band. Data were analyzed by one-way analysis of variance (ANOVA), followed by Duncan’s method for multiple comparisons (SigmaStat v3.5; SigmaStat, San Jose, CA, USA). *p* < 0.05 was considered significant.

## 5. Conclusions

The present study indicated that the marine-derived natural compound comaparvin obtained from *Comanthus bennetti* downregulates pro-inflammatory iNOS protein and gene expression in LPS-stimulated macrophage cells. We also demonstrated that carrageenan-evoked nociceptive sensitization behaviors (weight-bearing defects, thermal hyperalgesia, and mechanical allodynia) were significantly inhibited by systemic administration of comaparvin. Histological analysis and immunoblotting further demonstrated that carrageenan-induced upregulation of immune cell infiltration and expression of iNOS protein in inflamed paw tissues was inhibited by comaparvin. Future research can further explore the anti-inflammatory mechanism of action of comaparvin and its use in the treatment of inflammatory diseases. Comaparvin has a non-steroidal structure and could be developed into an anti-inflammatory and analgesic agent. We also discovered other compounds in *Comanthus bennetti*; we plan to perform further studies to assess their biological activity. Such studies and research on other marine compounds with similar structures could provide more chemical information on their anti-inflammatory activity; this information would be useful in the development of new anti-inflammatory and analgesic drugs.

## Supplementary Materials

Supplementary materials can be accessed at: http://www.mdpi.com/1420-3049/19/9/14667/s1.

## Supplementary Files

Supplementary File 1
