# Supplementary material for: Anti-Inflammatory and Analgesic Effects of the Marine-Derived Compound Comaparvin Isolated from the Crinoid Comanthus bennetti
Source: Molecules. 2014 Sep 16;19(9):14667–86. doi: 10.3390/molecules190914667 (PMC6271468; doi:10.3390/molecules190914667)

# Supplementary Information

**Figure S1.** $^{1}$H-NMR spectrum of comaparvin in aceton-$d_6$ at 400 MHz.

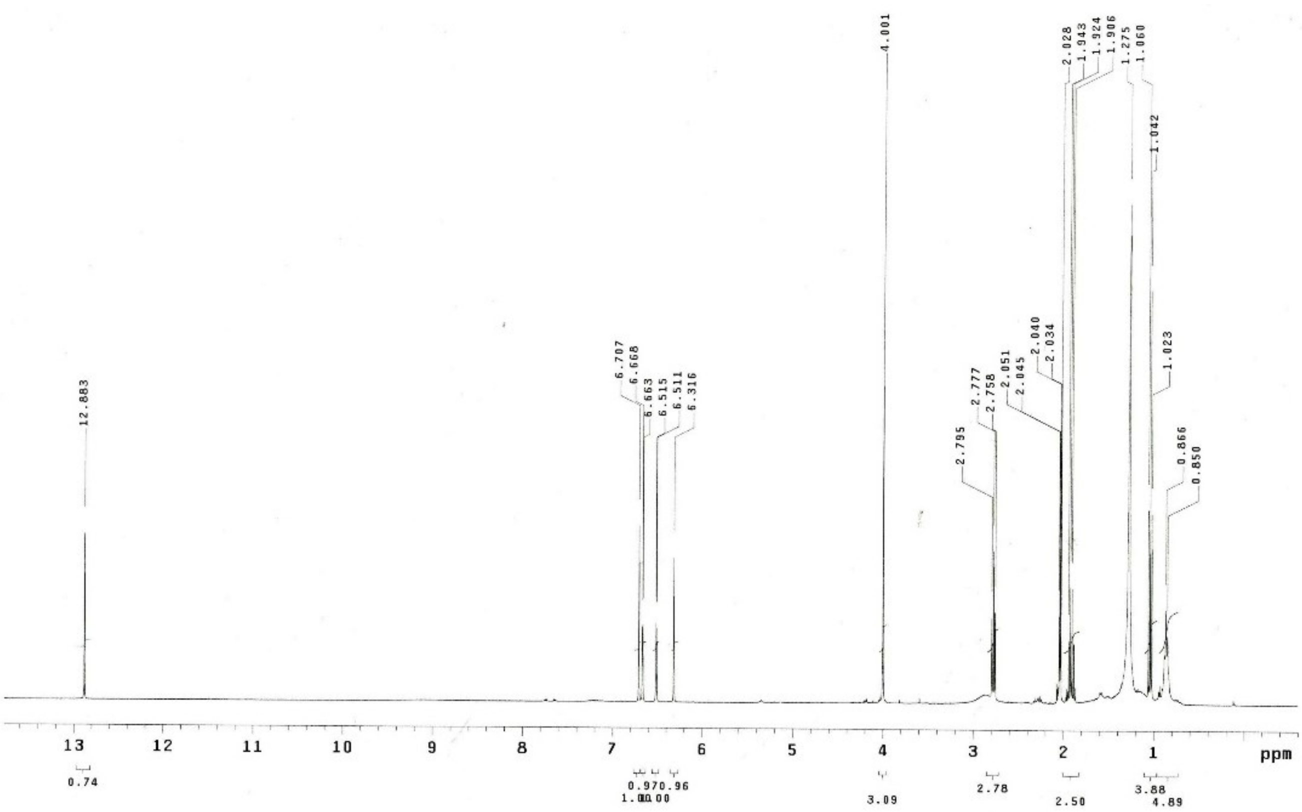


**Figure S2.** $^{13}$C-NMR spectrum of comaparvin in aceton-$d_6$ at 100 MHz.

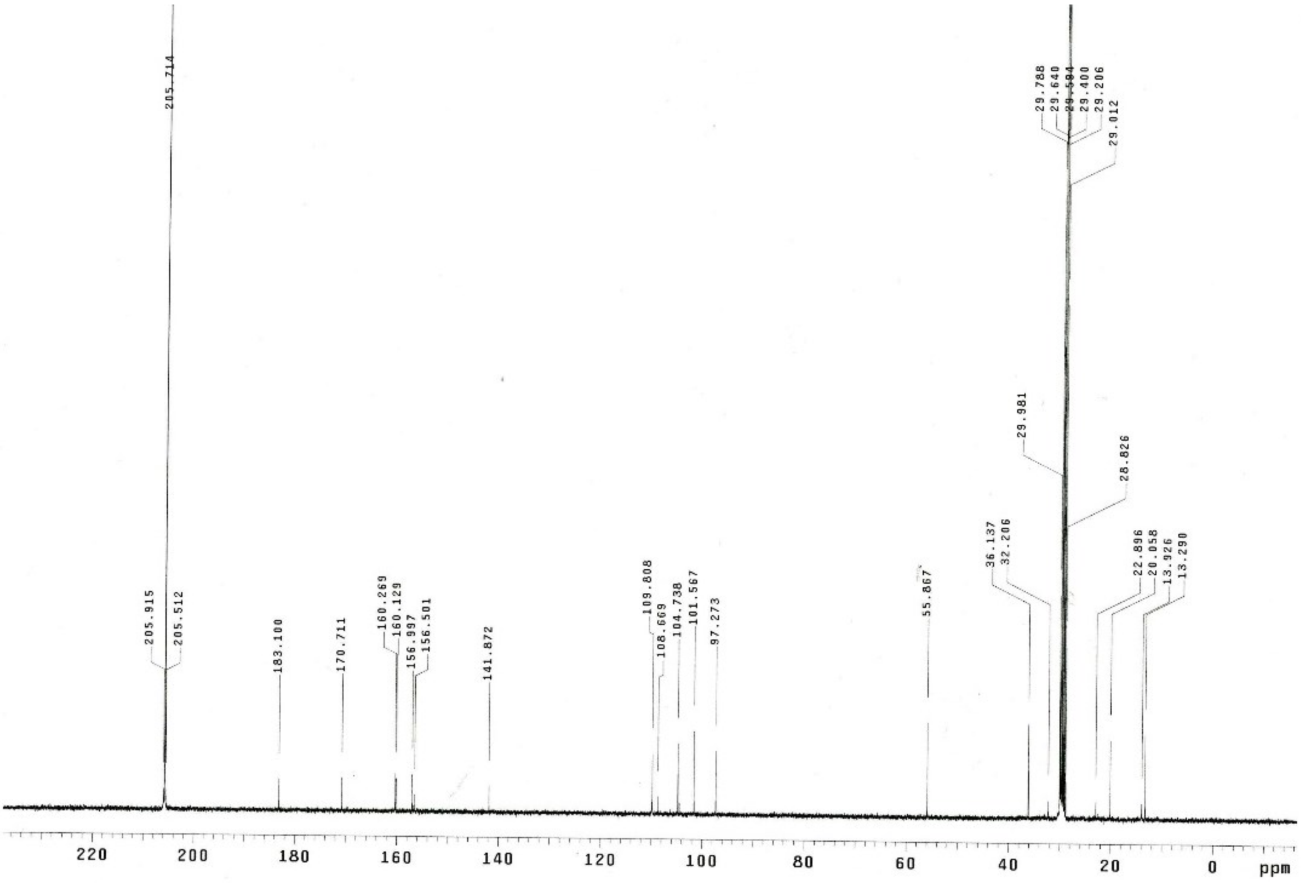

Supplement: Supplementary File 1 [file molecules-19-14667-s001.pdf]
